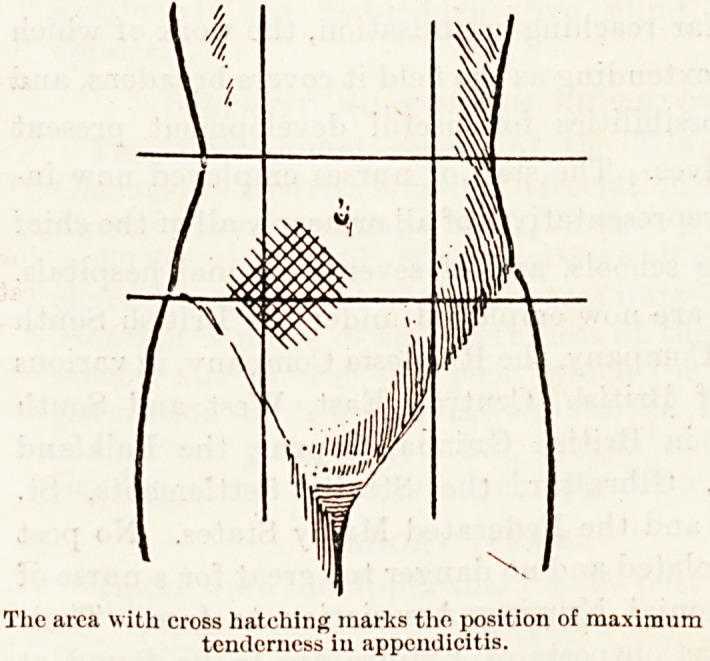# "The Hospital" Nursing Section

**Published:** 1906-07-14

**Authors:** 


### The Hospital.

**Nursing Section.**

Contributions for "The Hospital," should be addressed to the Editor, "The Hospital" Nursing Section, 28 & 29 Southampton Street, Strand, London, W.C.

No. 1,034.—Vol. XL SATURDAY, JULY 14, 1906.

### Notes on News from the Nursing World.

### REGISTRATION AND PARLIAMENT.

The Nurses' Registration and Qualification Bill, in charge of Mr. Claude Hay, is down in the Order-book for second reading in the House of Commons on Monday next, but there is not the remotest chance that it will be reached. Mr. Annan Bryce has given notice of his intention, whenever it comes on, to move "that it be read a second time upon this day three months."

### THE NEW HOME AT NEWCASTLE.

The Nurses' Home of the Royal Victoria Infirmary at Newcastle-on-Tyne, which the King and Queen formally opened on Wednesday, has been in occupation for a year. It is an oblong building of grey and red stone, with a ground floor and four stories. On the ground floor are the sitting-rooms for the home superintendent, sisters, and nurses, as well as the recreation room. These are well planned, with large bay windows. There are three wooden staircases between each floor, and a lift. Each nurse has a separate bedroom, with a long narrow window, fireplace, and furniture which is mostly fitted into the recesses. The corridors are of granite mosaic. On the top floor are the rooms occupied by, but not built for, the night staff, and unfortunately no precautions have hitherto been taken to deaden the sounds proceeding from the rest of the building. Electricity lights the whole place, and also supplies motive power for the lifts and the clocks. The spacious dining-room for the nurses is to be in the new hospital, which is connected with the Home by a magnificent conservatory.

### THE HOP-PICKING SEASON.

In view of the opening of the hop-picking season, and the numerous applications which we have had from nurses wishful to join in the work, we may state that there are now several agencies in the field. The Little Hoppers' Hospital at Five Oak Green, Kent, has been going on for seven or eight years, but a new institution is being opened at Golden Green, near Tonbridge, which the landowner is kindly fitting up for the mission. Both these hospitals, we learn from one of the organisers, have already a full staff. It is also proposed to open hospitals at East Peckham and Mereworth, but full details can be obtained from the Rev. F. G. Oliphant, Teston Rectory, Maidstone, who acts as secretary to the Church of England Hopping Mission. The Canterbury and Rochester Church of England Temperance Society appeals through its secretary, the Rev. J. Warner, from 64 Burgate Street, Canterbury, for funds, with which it is intended, if the response be sufficiently liberal, to employ a nurse or nurses on its own account among the hop-pickers. Of course it is well known that most of the ministrations of the nurses are amongst out-patients, as there are very few beds, but one of the most difficult questions is maternity work. This the nurses are not allowed to undertake. It is obvious that if expectant mothers imagined that they could depend upon any of the hoppers' hospitals to receive them, women who ought to stop at home would travel to the hop gardens and maternity cases would quite unduly tax the resources of the staff.

### COLONIAL AND BRITISH NURSING HOMES.

At the present moment the inhabitants of Melbourne are considerably interested in a controversy on the subject of nursing homes. The chief point of interest is the amount of fees which ought to be charged in the best private hospitals per week for the accommodation they provide. These range from three to six guineas per week, with an extra charge of three guineas per week for a special nurse. Only fully-trained certificated nurses are engaged in the private hospitals of Melbourne, and it is contended that special nurses should not in these circumstances be necessary if they entail an additional cost upon the patients or their friends. What the public need in Melbourne, as they need in this country, is a system which will provide the best hospital care for paying patients at an inclusive cost not exceeding three guineas per week. We have not yet found a solution to this problem in England, but a scheme of insurance is now being fully considered by experts, and there is some hope for a practical result which would give every satisfaction to all who are immediately interested.

### PUPIL NURSES IN FIJI.

Among the regular subjects which form the curriculum at the Colonial Hospital Training School for Nurses, in Fiji, are included readings from the Life of Miss Florence Nightingale, R.R.C., and instruction in the principles and methods of life assurance, with special reference to the Royal National Pension Fund for Nurses.

### SUGGESTED VISITING NURSE FOR HUDDERSFIELD.

A feature of the annual meeting of the Huddersfield and District Victoria Sick Poor Nurses' Association was an address from Miss Amy Hughes, who very wisely thinks that, as general superintendent of Queen Victoria's Jubilee Institute, it is not the least important part of her work to attend meetings in the provinces and afford them the benefit of her experience and counsel. We notice that Miss Hughes was impressed at Huddersfield by the number of operations which had been attended by the nurses, and that in her view they consisted of the class above the very poor, which ought not to have the services of the nurses without any return. The chairman's reply was that in respect to the operations the committee had asked the doctors to acquaint them with the cases in which the patients could afford to contribute to the funds of the Association; and if in such instances contributions are forthcoming, there is nothing more to be said. But, as the Association spent last year £97 more than it received, the financial position cannot be regarded as satisfactory; and in the event of difficulties the practical suggestion of Miss Hughes that a visiting nurse should be employed to attend certain cases merits the consideration of the managers.

### TRAINING AT THE NATIONAL HOSPITAL.

We are informed that the authorities of the National Hospital for the Paralysed and Epileptic, Queen Square, Bloomsbury, have made an arrangement whereby probationers will receive a three years' training, the second year of which is to be passed at a general hospital or infirmary. It is anticipated that this scheme will simplify the difficulties of those wishing to enter the nursing profession through the National Hospital, but who are deterred from so doing by the lack of general nursing experience to be gained; and also that it will be of advantage to those who desire to learn particulars of treatment by massage and electricity, because in exchange for the probationers, nurses will be received at the National Hospital, who during their stay will benefit by the special teaching. The lady superintendent will be pleased to hear from any matron of a general hospital who may be interested in this arrangement.

### BLACKBURN OPERATIVES AND QUEEN'S NURSES

Although at the annual meeting of the Blackburn District Nursing Association the satisfactory statement was made that the institution is now out of debt, Mr. J. P. Pollitt wisely emphasised the fact that the balance in hand of £244 in the general account, was mainly due to the transfer of £231 from the investment account. Mr. Pollitt went on to say that he was much disappointed that only about half the mills and workshops had responded to the appeal for help, the total sum being £238. He frankly told the meeting that unless the operative classes give from £500 to £600 a year the full staff of seven nurses cannot be retained. This is reasonable enough, and bearing in mind the popularity of the Queen's Nurses in Blackburn, it is not unduly sanguine to expect that by the end of another year the operatives will substantially augment their contributions.

### AMICABLE SETTLEMENT OF A LIBEL SUIT.

A dispute which threatened to be fought out to the bitter end was amicably settled at the Lincoln Assizes last week. The plaintiff was Miss Capper, a trained nurse, and the defendants were Miss Bromhead, superintendent of the Lincoln Nursing Institution, and the Corporation of Lincoln. Miss Capper accused Miss Bromhead of libelling her in certain letters in respect of the performance of her duties as one of the nurses at the epidemic of typhoid fever in Lincoln, and extended the suit to the Corporation because Miss Bromhead undertook the management of the nursing for them on the occasion of the epidemic. However, after Mr. Justice Walton had heard a good deal of conflicting evidence, counsel for the plaintiff announced on Friday that an arrangement had been arrived at, all charges and imputations against his client being withdrawn, and Miss Bromhead admitting that she had made them on insufficient and incorrect information. The judge, in expressing his satisfaction that a settlement had been agreed upon, said that he had heard nothing which seemed to have justified any reflection on the good intentions of Miss Bromhead in carrying on the most useful charitable work she was engaged in, and nothing which justified any imputation or reflection on the capability, qualifications, or conduct of Miss Capper.

### THE OUTBREAK OF TYPHOID AT BELMONT ASYLUM.

It was announced at the meeting of the Metropolitan Asylums Board on Saturday that arrangements had been made for the transfer of 62 patients suffering from enteric fever at Belmont Asylum to a portion of the Gore Farm Hospital. According to the report of the Asylums Committee it appears that since the outbreak the services of four additional male trained nurses and three female trained nurses have been obtained. No doubt the importance of engaging an adequate nursing staff was, in due time, brought home to the authorities, and it may at any rate be said "better late than not at all." Everything, it is asserted, is now being done, not only for the patients and staff, but to prevent the spread of the disease.

### GARDEN PARTY AT GUY'S HOSPITAL.

On Wednesday last week the annual prize distribution took place at Guy's Hospital. There was a very large number of visitors, most of whom arrived at 3 p.m. to witness the procession of the staff in their robes and to attend the presentation of the prizes by Sir W. Cameron Gull, who in a few graceful and suitable words congratulated the winners upon their successes. The whole hospital, schools, and Nurses' Home were thrown open, and throngs of visitors passed through them. The patients, especially the convalescent children, came in for a good share of attention from the guests. The band of His Majesty's 1st Life Guards played during the afternoon in the park. Tea was served on the newly-built terrace, and also in a large marquee and in the opposite quadrangle. The day was beautifully fine, and everything was at its best. At 5.30 "God save the King" was played, and the crowds began to disperse after many expressions of their appreciation and enjoyment.

### NURSES' ENTERTAINMENT AT ST. MARY'S HOSPITAL.

On Tuesday evening last week the nurses of St. Mary's Hospital, Paddington, invited the matron and sisters to a musical evening in the probationers' sitting-room, which was tastefully decorated with flowers. Several nurses sang solos, which were much applauded. The refreshments included strawberries, which a little party of nurses had purchased in the early hours of the morning at Covent Garden, and cream which was sent from Devonshire.

### THE DUCHESS OF MARLBOROUGH AND WEST HAM HOSPITAL.

Last week was one of quite exceptional interest to the staff at the West Ham and East London Hospital. On Saturday the matron, Miss Gough, held her annual "At Home," which was attended by about 500 guests, who inspected the hospital and enjoyed the entertainments provided. On the previous Wednesday the Duchess of Marlborough paid an unexpected visit to the institution at Stratford, bringing with her acceptable gifts for all the patients. The Duchess arrived during the patients' visiting hour, and not only spoke to each one, but also to their relatives. In many instances she most kindly arranged for patients to have a change of air in the country. She has also promised to give prizes to the successful candidates at the yearly examinations of the probationers. Nurses Adcock and Kerr will take the first and second senior prizes, and Nurses Austin and Wilson the junior prizes.

### ANNUAL REUNION OF NOTTINGHAM NURSES.

An interesting gathering took place on the lawn at Bagthorpe Infirmary on Friday last, when the members of the Parish of Nottingham Infirmary Nurses' League met from far and near for their annual reunion. A very pleasant afternoon was spent in renewing acquaintance with old friends. The staff had done their utmost to provide amusement by various sports, including tennis and croquet. The decorated bicycles, on which a great amount of time and trouble had been spent, were very good. The nurses' band also provided excellent music, tea was laid out on the lawn, and altogether the company had a most enjoyable time, good-byes being reluctantly said. Several who came from long distances spent the night in the Nurses' Home.

### ST. LUKE'S HOME FOR THE DYING.

On Tuesday afternoon last a meeting was held at St. Luke's Home for the Dying, to open a new ward for women. Some 50 or 60 guests were present, including many well-known London medical men. The wards looked very bright and pretty with large clusters of roses in every corner, the matron and nursing staff in smart uniforms, and the patients arrayed in all the glory of new pink bed-jackets. The undenominational nature of the Home was attested by several of the clergy and ministers. The beds in the new ward were already occupied, and if there were but more wards and money to maintain them they would be occupied too. Nurses, we understand, are not easy to obtain for this work, and it is quite futile for any but the most highly trained, keen, skilful, and vigorous women to attempt to undertake it.

### EXAMINATION AT EDMONTON INFIRMARY.

An examination of probationary nurses has just been held at Edmonton Poor-law Infirmary. There entered for the examination: Nurses C. Johnston, M. J. Gibson, E. C. Jones, A. Cottrell, F. D. Shilling, and A. Wright; and we learn that the six candidates passed very creditably both in the written and oral sections, Miss Johnston receiving the highest number of marks. The examiner expressed himself particularly pleased with the nurses' thorough knowledge of practical work. Nurses Johnston and Gibson have recently received the certificate of the Central Midwives Board after being trained in the maternity wards at the infirmary.

### THE COMMITTEE'S DILEMMA!

Amongst the "list of complaints" nursed by a parish nurse, and which had to appear in the annual report sent to all subscribers, was a case of spina bifida. During the nurse's absence on a holiday the Committee had to examine her returns, and the case of spina bifida nonplussed them. What could it mean? One and all agreed that the technical term used by the nurse must have a simpler meaning—a common or garden name such as the ordinary British public could understand. So with the interest of the uninitiated in view they put their wise heads together and the cleverest head among them, by getting to the root of things, settled matters to the satisfaction of the rest. Spina = thorn, Spina Bifida. = a complaint produced by the prick of a thorn. The result of this simplification was that in the list of patients whom the nurse had attended the one with spina bifida was entered in the report "Gathering from a thorn." When the nurse, on her return, read the report, she suggested—not to the Committee—that spina bifida deserves a place with lex talionis, sarcophagus, and other standard definitions.

### PROGRESS AT LOWESTOFT.

The Lowestoft Guardians, acting upon the strong recommendation of the medical officer, have agreed to appoint another assistant to the nurses. How essential this assistance had become may be gathered from the fact that in the infirm wards of the workhouse there has been a single night nurse on duty, with 44 inmates to look after. Of these nearly all are bed-ridden, five blind, and one epileptic.

### DISTRICT NURSING IN KINGSTOWN.

The first annual report of the Kingston and Glenageary District Nursing Association shows that during the year ending June 1, 73 cases were nursed and 2,308 visits paid. The cases were all of a serious character, and a list of the diseases nursed is given. Mention is made of the usefulness of the Nourishment Fund for the sick poor, which the nurse, by permission of the Committee, started in March. Numerous gifts of clothes, linen, and knitted bandages have also been much appreciated.

### SHORT ITEMS.

There were 130 applicants for the post of matron of Walsall and District Hospital, to which Miss Gaved Wills, lady superintendent, of Newark Hospital, has just been appointed.—A concert in aid of the Trained Nurses' Annuity Fund was given, by permission of Lord and Lady Ellesmere, at Bridgwater House, on Tuesday afternoon, and the large company included Princess Christian and other members of the Royal Family.

### The Nursing Outlook.

"From magnanimity, all fears above;
From nobler recompense, above applause,
Which owes to man's short outlook all its charm."

### "OUTPOSTS OF EMPIRE."

Ten years ago there was practically no adequate, or indeed any nursing properly so-called, in most of the smaller British colonies and dependencies. Lady (then Mrs.) Piggott, with commendable courage and energy, took up the question, and mainly by her efforts the Colonial Nursing Association was established to provide trained nurses for the British colonies and dependencies and for British communities abroad, both for private and hospital work. Mr. Chamberlain, when he became Colonial Secretary, cordially supported Mrs. Piggott in her efforts, with the result that more practical work of a really valuable kind has probably been accomplished in ten years on Mrs. Piggott's initiative than by any other similar organisation in the same time. Commencing with six nurses in 1897 the Association is now in charge of a staff of 305 nurses, of whom 215 are in Government and 90 in private employment. Several of the nurses have belonged to the Association for eight or nine years, and have in the case of some of them completed as many as three engagements either in the same colony or in different parts of the world.

The tenth annual report (1906) just issued testifies to the great need there was for this Association which, from the humblest beginnings, has grown into a far-reaching organisation, the work of which is ever extending as the field it covers broadens, and new possibilities for useful development present themselves. The staff of nurses employed now includes representatives of all or nearly all of the chief training schools, and of several colonial hospitals. Nurses are now employed under the British South Africa Company, the Rhodesia Company, in various parts of British, Central, East, West and South Africa, in British Guiana, Cyprus, the Falkland Islands, Gibraltar, the Straits Settlements, St. Helena and the Federated Malay States. No post is too isolated and no danger too great for a nurse of the Colonial Nursing Association to face. Their nurses as outposts of Empire are to be found at Zungeru and Lokoja, at Entebbe, Kuala Lumpur and Seremban, names which few or none have ever heard of before, and whose situation on the face of the globe is unknown to the majority of people. At Bangkok, in Ceylon, at Costa Rica, Hong Kong, Tokio, Singapore, Teheran, Zanzibar, on the Gold Coast, in Northern and Southern Nigeria, Kandy, and Terak, to mention a few of the more active centres, and even in places like Oporto and Venice, the Colonial Nursing Association's nurses are now employed to the great comfort and cheer of British residents. It is a truly wonderful organisation, and the more closely the work is examined into, the more deeply it must impress every one, who has sufficient imagination to fill in the details and grasp the immense value of the labours of the isolated but devoted women, who constitute the nursing staff. All honour to them and special honour to Lady Piggott, who must indeed be proud and thankful to realise that her idea devotedly pursued has grown into one of the most fruitful organisations in the British Empire.

It has been the wise policy of the managers of the Colonial Nursing Association to secure the co-operation of the two great schools of tropical medicine, who have gladly received nurses on joining the Association, and have given them three months' training in tropical diseases free of all cost. Nurses are further helped by the payment of fees for special training, and by providing them with outfits for service abroad. It is the practice of the Association to make advances for the outward passages of nurses, nearly £350 having been expended in this way last year, and the committee have always found that the nurses repay such loans with great punctuality, well within the time limit, usually one year. By making grants to small British communities, the Association promotes the development of the work, for, as one such community puts it, "it is so encouraging to workers in this outpost of Empire, to feel that there is some one taking an interest in them at home." The ideal of the Association is to have at least one nurse, and, should the circumstances and funds permit, a hospital with a trained staff, wherever the climate is most deadly and the work most lonesome. It is well contended that wherever disease is most rife, there skill should be found to fight it to the death with every aid which the most recent science can suggest. Such is the work of the Colonial Nursing Association, and the constant endeavour is to secure that nothing shall anywhere be lacking which can be provided to help to maintain or restore health and to protect the lives of those who go forth so bravely to serve the British Empire in the uttermost parts of the earth. We who remain at home can do our part by becoming subscribers to or collectors for the Colonial Nursing Association. Its funds are none too plentiful, the work is always extending, and money given to it will be well dispensed in making the lot of England's sons happier in their days of sickness and trial. We owe it to ourselves to lend an intelligent hand to this bit of Empire building.

### Abdominal Surgery.

By Harold Burrows, M.B., F.R.C.S., Assistant Surgeon to the Seamen's Hospital, Greenwich, and to the Bolingbroke Hospital, Wandsworth Common.

### APPENDICITIS.

The vermiform appendix is a slender hollow diverticulum which opens into the cæcum close to the ileo-cæcal valve (see illustration on page 146). It is about three and a half inches in length, is lined with mucous membrane similar to and continuous with that lining the cæcum, and has an outer covering of peritoneum.

In some animals—the rabbit, for example—the cæcum and appendix are relatively enormous, and play an important part in the process of digestion. In man, however, the appendix is of little or no value to its possessor, and people do not suffer any apparent disadvantage from having the appendix removed by operation.

Inflammation of the appendix, or appendicitis, is one of the commonest of human ailments. And it is probable that few individuals pass through life without having one or more slight attacks; although these are apt to pass unrecognised.

Symptoms due to inflammation of the appendix frequently are attributed to some other than their true cause. So-called bilious attacks, especially in children, are in many instances appendicitis. And not rarely the vomiting and stomach-ache that are regarded as the results of dietetic indiscretions, such as the eating of unripe fruit, are really due to inflammation of the appendix. Such mistakes are less likely to be made if their possibility is borne in mind. Even apart from the question of proper treatment, the matter is important, both because experience shows that a person who has had one attack of appendicitis probably will have others, and because in eliciting the history of a case supposed to be appendicitis it is necessary to inquire whether the patient has had any past illnesses which might be regarded as previous manifestations of his present complaint.

### Causes of Appendicitis.

Rarely the appendix is affected with cancer, actinomycosis, tuberculosis, or typhoid ulceration. In other cases a foreign body, such as a pin or a small shot, becomes lodged in the appendix and brings about inflammation. But these are not the common causes. Usually inflammation of the appendix is due to infection of its mucous membrane by micro-organisms derived from the bowel.

The results of such infection vary. There may be merely a temporary swelling and redness of the mucous membrane, which soon subsides. There may occur ulceration which heals and leaves some narrowing or complete occlusion of the canal of the appendix. In yet other cases the ulceration extends through the entire thickness of the walls of the appendix so that the peritoneal cavity becomes infected. In other cases the infective process leads to gangrene of the appendix. The chief danger in all cases is that the infection may spread through the walls of the organ and cause peritonitis.

The peritonitis once started may spread with great rapidity through the abdomen and lead to a fatal issue within 48 hours. In other cases the peritonitis remains local, and either an abscess is produced or the inflammation subsides without the formation of pus, the only gross result being the formation of intraperitoneal adhesions. If an abscess forms, and the case is neglected, the pus may in course of time become absorbed, or it may increase in quantity and burst through the abdominal wall, or open into the bowel or bladder or general peritoneal cavity. In yet other cases pyæmia results, as with neglected abscesses in other parts of the body.

If the case resolves without worse result than the formation of adhesions, these adhesions may sooner or later produce intestinal obstruction; they are very likely to cause constant or frequently-recurring pain, and they greatly add to the difficulties and dangers of a subsequent operation for removal of the appendix.

From this catalogue of complications some idea may be formed of the penalties of neglect and delay in appendicitis.

On the other hand an operation skilfully performed within the first 12 hours of a first attack is nearly sure to cure the patient and relieve him of the dangers of subsequent troubles. At present, however, public opinion is not quite educated up to this line of treatment, nor are the means always available for a proper operation at such short notice, and so the general rule is to operate at once only in very acute cases, and in others to await developments and hope for the best.

### Symptoms.

In a typical case the principal symptoms are pain, tenderness, and rigidity in the right lower half of the abdomen, associated with a raised temperature,

The area with cross hatching marks the position of maximum tenderness in appendicitis.

increased pulse rate, vomiting, and constipation—the symptoms, in fact, of a local peritonitis. But cases vary a good deal in their manifestations, and sometimes an abscess may be present with hardly any symptoms at all. If the peritonitis spreads to the bladder and rectum, there will be frequency of micturition and pain during the act, and pain on defæcation with the passage of mucous and perhaps blood by the rectum, and the administration of an enema may cause severe distress. These are points which the nurse should always be careful to observe and report to the surgeon. As in other abdominal disorders, specimens of vomit, urine, and bowel evacuations should be preserved for subsequent examination and analysis.

TREATMENT.

If immediate operation is not decided upon, a large responsibility will rest upon the nurse in charge. Not only will she have the special and routine nursing of the case, but in addition she will have to keep a careful and constant watch for the earliest signs of any complication, and if any arise to report them at once to the surgeon, so that no valuable time may be lost. At the same time she should use especial care not to render the patient fidgety, anxious, and apprehensive. It is a little difficult to explain how this is to be avoided, but everyone of experience knows how quickly and completely a patient can be unnerved by the fussy nurse who rushes about the room, whose movements seem to be all by jerks, and who speaks hurriedly and in a high-pitched key. Perhaps it is unnecessary in these days of nursing proficiency to labour the matter, but it is of so much importance that a reference to it seems to be justified.

The signs that must be regarded as of serious significance are rising pulse-rate, vomiting, increase of abdominal pain, rigor, distension of the abdomen, and shock. Any one of these ought to be reported without delay to the medical attendant.

The medical treatment of appendicitis consists of absolute rest in bed, with ice or fomentations applied to the abdomen, and a very light liquid diet. The lower bowel should be emptied by means of an enema. If there is much vomiting it may be necessary to withhold all food by the mouth, only sips of water being allowed. To ease the pain, which is the most prominent symptom in most cases, opium or morphia is occasionally given, and especially if the patient is very restless. But these drugs are apt to obscure the onset of complications, and for this reason the greatest caution is required in their administration.

### The Nurses' Clinic.

### INFLAMMATION.

In the long catalogue of diseases requiring a nurse's attention comes most frequently, perhaps, "inflammation." In the hospital, on the district, as a private nurse, she is perpetually confronted with its ravages. It may be surgical—appendicitis, peritonitis, abscess, sprain, etc.; or it may be medical—pneumonia, pleurisy, bronchitis, laryngitis, etc. It is such a common, every-day event in a nurse's life that she carries on the treatment ordered without much thought, and is somewhat puzzled if asked "what to do" or "Why is so and so done?" in any case where the symptoms point to inflammation. That treatment is most essentially a medical man's province is undoubted, but an intelligent carrying-out of his directions can only be achieved by one who understands the reasons for the various principles of treatment. When there is no reason to expect inflammation certain symptoms should make a nurse suspicious—heat, redness, pain, swelling, or any loss of power in any part of the body. Heat may be felt if it is surface inflammation, or shown by rise of temperature. Pain varies in intensity and in character, and is therefore generally indicative of the part affected and also to some extent of the progress already made by the disease or injury causing inflammation. Accuracy in a nurse's report when sending for a medical man will ensure his being fully prepared when he comes, and early treatment in any inflammation is most essential. The first stage in inflammation is overfulness and consequent dilatation of the blood-vessels; secondly, obstruction; thirdly, exudation. In the third stage pus is formed, and early treatment may avert the formation of pus or at any rate prevent the further disaster of ulceration.

All treatment is summed up under the following heads: (1) remove the cause; (2) rest the inflamed parts; (3) reduce the blood-pressure; (4) prevent fresh infection.

1. This rests almost entirely with a surgeon or physician and may mean the opening of an abscess, removal of a foreign body, or treatment by drugs or antitoxins.

2. But after the opening of the abscess or removal of the foreign body, nursing as well as treatment is required. Abscess in the breast will call for support of the breast by bandaging. If a foreign body is removed from the eye, the eye is rested by covering it with a pad to prevent friction of the eyelid or the effort to see things. Injured limbs may require splints and sandbags. Appendicitis and all the most severe forms of inflammation, medical or surgical, call for complete rest in bed and most careful nursing. Whether the rest is complete or partial, nursing is much required under this second head of treatment.

3. And also in reducing the blood-pressure. This is done by heat or cold. Hot applications dilate the blood-vessels, stimulate the tissues, and send a greater flow of blood to that part. Cold has the reverse effect—it contracts the vessels, lessening the blood-flow, and lowering the vitality. Therefore in the early stages of inflammation, before exudation is begun, cold is most useful. It should not be applied after suppuration is established as it lessens the vitality, nor should it be used for old people, as age predisposes to a want of vitality. If cold is ordered it must be continuous. If there is an interval between the cold applications, the white corpuscles may escape, an abscess form, and the lowered vitality caused by the cold applications, or at any rate greatly increased by the treatment, will render healing a very slow process. Cold compresses must be constantly changed and where possible ice kept in the water, one compress being always in the water. Heat of the hand in wringing them out takes away from the coldness, so that the water cannot be too cold. Eau de Cologne or ammonia will keep water cool, and serve as an evaporating lotion. An ice poultice is sometimes ordered and is made of chopped ice sprinkled with salt, laid between layers of linseed meal or bran and put into a gutta-percha or mackintosh covering. The salt delays the ice melting and the bran or linseed absorbs the moisture. When put into the gutta-percha the edges are fastened together with heat (a lighted match), spirits of chloroform, or turpentine, or sewn together. In an emergency a sponge-bag is useful for an ice poultice or to make an ice-cap. Ice for an ice-cap should be broken in very small pieces and a little kitchen salt added. The cap should be suspended so as to rest lightly on the head. If

**THE NURSES' CLINIC—*Continued.***

ice bags or poultices are ordered for pericarditis, they should never do more than rest very lightly on the heart, otherwise they will greatly add to pain; a cradle is used in hospital, but in private houses a chair or a card box may give the necessary support. The effects of cold must be carefully watched by the nurse. Hot applications relax the vessels and lessen pain. They increase vitality and hasten the formation of pus. For wounds, lint wrung out in hot water or an antiseptic solution is best. An abscess should not be allowed to burst into a linseed poultice. Hot-water fomentations are often used for the throat, being less bulky. In abdominal inflammation hot fomentations are often preferred to poultices, being lighter and more comfortable; the only drawback to them is that they require constant changing, for they do not retain the heat very long. Linseed poultices assist the solid lung in pneumonia to "resolve" or free the air-cells blocked with inflammatory products. In "dry" pleurisy hot applications retard the formation of lymph. Hot steam reduces the swelling in laryngitis or bronchitis due to the inflammation of mucous membrane. Linseed poultices should be light, well mixed, and not too dry. Mustard is added according to the age of the patient and also with due regard to the frequency of the application. Charcoal added to linseed, 1 to 4 parts, is sometimes ordered for foul ulcers, but antiseptic fomentations are better. Starch poultices are very soothing in many skin affections.

4. In surgical cases the wound must be kept clean and all antiseptic precautions used with regard to the hands and dressings. In medical cases, rest aids in preventing the spread of infection to another organ.

### Incidents in a Nurse's Life.

### IN A PRIVATE ASYLUM.

I had had a very hard time. After having finished a three years' training in a large provincial hospital, I had been sister of a women's ward and theatre for some time; then accepted the post of night superintendent in one of the largest Scotch hospitals, and so was naturally very pleased when I was asked to offer myself as a candidate for the matronship of a high-class private asylum. A friend of mine was holding the post, and was leaving for promotion. Knowing my anxiety to get off night work, and also that I was ambitious for a matronship, she had kindly mentioned me to the medical superintendent, who was glad to have a suitable candidate specially recommended.

After a successful interview I was delighted with my luck, and felt sure that, in contrast to my previous hard work, life in an asylum would be a bed of roses, and I looked forward with pleasure to the experience. A general feeling of satisfaction came over me when I arrived to take up my duties. The building was situated in large and beautiful grounds, the rooms were elegantly furnished—quite unlike an institution—and there was an air of comfort and grandeur everywhere around. The doctor was good and kind, and the nurses thoughtful, and the patients gave me a hearty welcome. For the first week I enjoyed the novelty, but the second week a member of the staff, whom I had found to be my best help (unaccustomed as I was to the care of insane patients), came and informed me that she had also been appointed to a matronship, and would be glad if I could let her go almost at once. To me this seemed almost an impossibility, as she had been in charge of one of our worst cases for some four or five years, and no one else had the least control over the patient.

However, the difficulty had to be faced, so I began to wonder how. I remembered that my sister, who was also a trained nurse, might like the experience, at any rate for a time, so when I mentioned it to the superintendent he was pleased with my suggestion, and sent for her immediately. Unfortunately the patient seemed to know that her "attendant" was leaving, and was in a most excited state. In fact she was more like a wild beast than a human being, much less a refined and educated lady, and on my entering her room with my sister she sprang upon her, and would have seriously hurt her had I not been there to protect her.

Bedtime came, and as the "attendant" (called by courtesy her companion) refused to remain alone, I decided to stay with her. So we both lay down on an unoccupied bed in the same room, listening to the groans and coarse language of the patient, who was on another bed, when suddenly she sprang up and began to heap fearful and disgusting epithets on our heads, and to run about the room. Then she got to blows, and although she was only a small woman and we were both fairly tall and strong, she almost overpowered us. We had a fearful struggle for over an hour; indeed until we were nearly exhausted—but the patient seemed as full of energy and strength as ever. Finally I decided that I must get some help, and called up two of the ordinary nurses, but it was not until all four of us had battled with her for some time that we got her under control. She was for a considerable period growling and snapping like a dog, and it was dangerous to go near her for some days. As our institution was not well adapted for such violent cases, she was removed later to a neighbouring asylum and isolated.

I often think of my first experience with a lunatic, and I have come to the conclusion that the nursing of such cases must be a special gift, and cannot be lightly undertaken, even by those who have had good general training. Moreover, the women who devote themselves to such trying and arduous duties are worthy of the highest praise. Certain it is that every year they are more appreciated by the public and by nurses generally as they deserve to be.

Although I consider the sixteen months' experience I gained was very valuable, I must admit that I was only too pleased to return again to my general hospital work at the end of that time, and to resign my position in favour of one who, like me, was expecting to find life in an asylum "a bed of roses."

### Home Preparations for Intending Probationers.

A girl who has a nurse's career before her vision, but is debarred for the time being on account of being too young, or from some other cause, from entering the profession, can do numerous things by way of preparing herself at home.

*Health.*—This is the first consideration. We must suppose that she is strong and has a good constitution. But if this is neglected and bad results follow it is no use thinking of nursing. She must look on this as an immutable rule, that if not well herself she cannot do justice to her patients. Her teeth must be put in perfect order, which may ultimately save endless gastric trouble. Another reason why this must be done is that in all probability it will be the first question she will have to answer in her application form.

*Method.*—She must train herself to be methodical in her home duties, neat and orderly in her bedroom. Moreover, if she sees anything which needs doing, she must do it immediately and thoroughly, and so make it a practice, remembering that use is second nature. She should also try not to be self-conscious. A self-conscious nurse is an abomination to everyone. Under any and every circumstance she must endeavour to keep cool and not to get flurried or excited.

*Discipline.*—If it is her lot to have young children as members of her family, she should study them carefully, learn how to wash and dress them properly, how to be firm and kind but yet make them obey, to say a thing and mean it, and let them see that she does.

*Punctuality.*—She should be down to breakfast at the proper time, not five minutes late. If punctual herself and showing that she expects others to be, it will be a good step gained.

*Thoughtfulness.*—This may be exercised daily, by respecting those over her, more especially the aged. Have they not trodden life's thorny path and become experienced in parental trials? If they appear unnecessarily fidgety she should still try and be patient, thus developing another virtue which a would-be nurse is bound to possess.

*Cooking and Housekeeping.*—The girl should acquire an elementary knowledge of cooking and housekeeping. It will prove useful both in hospital and private work. She must not imagine that the latter will be all poultice-making, taking temperatures, and making beds. The first part of training will most probably be spent in scrubbing mackintoshes, sweeping and dusting, brass cleaning, etc. These duties are sometimes given to see what a girl is made of.

*Observation.*—It is easy with a little thought to cultivate powers of observation. This can be very practically carried out in daily life. For instance, while out walking take in with the eye everything that comes along. Notice the policeman on his beat, his eyes are everywhere and on everyone, observing the minutest details too. When anything has been noted specially, detail the episode accurately, state the fact only, not what ought to have happened, and if the event is narrated more than once do not add to it each time. This is such an easy thing to do, and when in hospital frequently causes much harm. A doctor wants to know just what has taken place during his absence, not what ought to have occurred. Thus truthfulness is another virtue for a nurse.

*Needlework.*—Dressmaking would be no disadvantage, for a nurse often has to mend and make her patient's garments.

*Sanitation and Ventilation.*—These should be thoroughly understood and carried out systematically at home.

*Flowers.*—Let home be made to look bright with flowers and plants. If arranged daintily these form the best addition a room can possibly have, but if all are crowded tightly in one vase they look irritating and anything but effective.

Always wear a bright face, and try to cast a ray of sunshine wherever you go; a patient feels quite "down" enough without having a sad face to look at every time a nurse appears at his bedside.

*Other Talents.*—If she is anything of a musician a would-be nurse should not allow her talents to lie dormant. It is the greatest mistake to imagine that because a girl thinks of becoming a nurse she must give up all kinds of recreation. A bright, sensible, accomplished girl will be both welcome and useful in either the hospital ward, hospital home, or private nursing.

*Studying.*—It is not necessary to do much cramming before entering hospital; in fact, I think that ignorance is bliss for the time being, unless a girl has to pass a preliminary examination. Then she will, from looking over the subjects in which she has to qualify, at once see the best books to study. Otherwise her spare time may be spent in reading any sound moral books. She would also find it interesting to take in one or two good nursing journals. It would give her a very good insight into the nursing world.

*Outfit.*—Have a good stock of underclothing, and have everything plainly marked—initials are of no use—or it will be found in a very short time that the stock has greatly diminished, to the benefit of that terrible lady—the laundress. Different hospitals have different rules as regards uniform, so she must act accordingly when these are known. No jewellery will be required in the outfit, so that can be left at home before starting. The intending probationer should be very careful not to take too many knick-knacks; most probably she will have to dust and keep her own room in order, so this will be a great consideration.

We will suppose that by this time everything has been satisfactorily prepared. Her next step is to look up the best training school. No doubt her family physician will advise her on this matter. She must then write to the matron of the selected institution, and ascertain if she has a vacancy for a probationer, giving her full particulars as to age, height, health, etc. She must not look forward to an easy or happy time for the first three months; but if she perseveres and is really in earnest, no life could be spent more pleasantly, honourably, or profitably than in doing what she can for those who are sick, afflicted, and helpless.

### A Night with the Night Sister.

As the wardmaid noisily dragged a chair across the floor and deposited a cup of tea upon it, the night sister woke with a start. "It's eight o'clock, sister, and I've brought your hot water." "Yes, thank you, Mary," said sister sleepily, much inclined to drop off again; but outside the bell clanged persistently, and a glance at her watch showed her it was already two minutes past eight, so she had only five delicious minutes in which to get thoroughly awake before rising. It was 8.29 as she descended the stairs into the big dining-hall, where nearly fifty night nurses were assembled. She had paused before entering for one or two stragglers who were heard hastening along the passage, then, as soon as grace was said, breakfast began. During the meal two probationers entered somewhat breathless.

"Please, sister, I was ready, but I had to go back for my night shoes."

"I never heard the bell, sister, and did not wake till nurse called me."

"I am sorry, nurse, but I cannot take such excuses, and the bell went as usual."

They thought her hard as they took their places, and sister half sighed as she noticed their sullen looks, and wondered if they thought she took a delight in marking those hateful "lates" in the book.

Presently all rose, grace was said, then the names called, and all filed out to chapel. A probationer stopped and said timidly, "Nurse Cook has not slept to-day, sister, and her throat seems bad." "Thank you, nurse, I will go and see her," said sister, thinking hard at the same time who of the "extras" could best fill her place as head nurse in the large female medical ward. Immediately after chapel the decision is made: "Nurse Pole go to 'Mary,' please, to do Nurse Cook's work, and Nurse Tillet go to 'Vanderbilt' for the present."

Having settled the destination of the "extras" she is about to go to the Nurses' Home to see the sick nurse when the day sister of the casualty ward enters the office.

"Oh, good evening, sister; I came to ask you whether you would mind taking an operation for me to-night as my head is so bad that I want to go to bed early."

### A NIGHT WITH THE NIGHT SISTER—*continued.*

"Certainly, sister, of course I will; what is the case?"

"Laparotomy, if it is done," significantly, "Mr. Fitt is coming about a quarter past ten and will decide then if he will operate, but the man is so bad he may not do it. Well, thank you very much, sister, the probationer will come and tell you when he arrives. Good night."

"Good night, sister, I hope your head will be better to-morrow," says the night sister, who has already calculated that it will be quite 10.30 before the operation is begun, by which time her surgical colleague will be on duty and able to take the case.

Another knock is heard and the sister of the twin wards —"James" and "Marion"—enters.

"Good evening, sister, I came to ask you if you could spare me an extra for an hour or two in "James." Nurse is very busy and I hear there is an intestinal case in the surgery likely to come to us any minute."

"Yes, certainly, sister, I can send an extra at once. How is No. 11, the pneumonia who was so bad last night?"

"Better, decidedly better, but another one came in to-day who is as bad as he can be, then there were four operations and the typhoid is quite delirious, so you see nurse has her hands full. Well, thank you, sister, I shall be glad of the extra" and she hurries back while the night sister unearths Nurse Tillet from "Vanderbilt" who, having just settled down to pad a Thomas's splint, departs with mixed feelings. Up, then, after leaving a note on the slate as to her whereabouts, to the top of the home to see Nurse Cook. Undoubtedly a nasty throat, and temperature 100°. After doing what she can for her, she finds the home sister who promises to take her some milk and a gargle and to look in the last thing.

Upon returning to the office she is not surprised to find on her slate, "Mr. Isaacs is in 'James,' operating." The patient is just being placed on the table as she enters, the preliminaries are quickly performed, and for the next hour nothing is heard but the rattle of steel instruments, the stertorous breathing of the patient, and an occasional brief request for lotion or sponges. Presently the anæsthetist asks sister to give the patient strychnine hypodermically, the surgeon half pauses in his work, looks fixedly at the man. "Transfuse him," he says shortly, and two students spring to obey. The operation is now quickly finished. "Gauze please, wool, bandages, and thank you," and then the man is carried back to bed with the transfusing apparatus still *in situ.*

Half an hour later sister is able to begin her rounds. The first two wards are all quiet, no fresh admissions, and no really serious cases. On entering the third ward "Esther" nurse rises to meet her with trembling limbs and flushed, weary face.

"I'm afraid you are not well, nurse," says sister sympathetically.

"I feel better than I did, thank you, sister; but my back and legs ache so dreadfully. I am afraid it is a touch of influenza."

"Have you taken your temperature?"

"No, sister, I don't believe I have one"; hesitatingly, "I don't feel much like one."

Sister has so often heard this before that she merely hands her a thermometer with a smile, and says, "Well, sit down and take it now. No, don't come round. I'll go alone. Everybody seems very quiet."

The thermometer registers 103°, and nurse then confesses to headache and sore throat as well. Something must be done. The probationer is very steady, though not senior. Sister cogitates deeply for a minute, then says, "Are all the night medicines given?" "Yes." "Then you must go straight to bed. Nurse thoroughly knows the ward routine, and I shall look in as often as possible and give her help in the morning." Arrangements completed, "Mary" is the next ward. Nurse Pole is somewhat harassed by her duties, and there are various points to be straightened out, a nasal feed to be given and a new admission to be seen and entered in the books. In the gynæcological ward nurse wants help with two refractory patients. In the maternity ward there is a new baby, over whom sister would fain linger, but she has now to hurry down to superintend the last of the "meals," which is served for the nurses in the dining-hall, after which her colleague and herself are able to sit down and discuss cold beef and pancakes.

It is now past 2 A.M., another visit to the sick nurses upstairs, then a message arrives from "James" to come at once. The newly-operated on patient has suddenly collapsed and is sinking fast, and dies a few minutes after sister enters the ward. The usual notices and telegrams are written, and after a careful look round at all the other patients she returns to the office for a brief rest before starting the second round.

Nothing particular happens on the next round. A bad heart case wishes to see the priest, so sister has to ring him up; otherwise the wards seem to have settled down. Another visit to administer a gargle to the two sick nurses, and then back to the office for a cup of tea and a peep at the newspaper. Between five and six life begins to stir again in the hospital. Dustmen appear out of the gloom with their unsavoury burdens, sleepy wardmaids arrive with pails and brushes to clean passages and offices, and the night sisters consult their watches and hurry away to their respective divisions. The sick nurses are first seen and attended to, and then begins the third and most cheerful round. What a different aspect the wards now wear! The patients are all refreshed by the night's rest, and some are busy washing, some breakfasting, and some convalescents cheerfully assisting nurse by carrying away empty plates and mugs. One girl who looked ghastly in the shaded night light now emerges fresh and radiant, with blue ribbons in her hair; a little four-year-old calls out gaily, "Dood morning, sister," and holds up her newest toy for inspection; the babies are being clad in pink gowns, and their elders don flannel jackets while comparing notes as to their night's sleep.

In the male wards the men are anxiously awaiting the newspaper boy, and are all eager to give sister the very latest news or their opinion on the fiscal policy; the barber goes from one to another performing his mysterious rites.

One old Daddy asseverates solemnly that he never closed an eye, but this daily statement is received with polite incredulity as he declares in the same breath that he never saw nurse or sister all night. The typhoid in "James" even conjures up a ghost of a smile, and takes his milk without a murmur. Everyone is better, and it is with a light heart that sister finishes the last round at ten minutes to eight, and then finds time for a last cheering word or two to the sick nurses.

### To Nurses.

We invite contributions from any of our readers, and shall be glad to pay for "Notes on News from the Nursing World," "Incidents in a Nurse's Life," or for articles describing nursing experiences at home or abroad dealing with any nursing question from an original point of view, according to length. The minimum payment is 5s. Contributions on topical subjects are specially welcome. Notices of appointments, letters, entertainments, presentations, and deaths are not paid for, but we are always glad to receive them. All rejected manuscripts are returned in due course, and all payments for manuscripts used are made as early as possible after the beginning of each quarter.

### Association for Promoting the Training and Supply of Midwives.

A PLEASANT little function was planned to take place last Wednesday, under the auspices of the Association for Promoting the Training and Supply of Midwives, at 9 Airlie Gardens, Campden Hill, the residence of Mrs. Wallace Bruce. Invitations had been sent to all the midwives trained or in training by the Association, and badges were to be distributed by Lady Balfour of Burleigh to those who had completed six months' satisfactory work. Very effective and pretty are these little silver badges, in the shape of a Maltese cross, having on one side the words Love, Truth, Pity, Mercy, and on the reverse the title of the Association.

It was fortunate that none of the twelve midwives who had earned the badges were able to be present to receive them. Most of them, it was known, were prevented from coming by urgent engagements, but it was quite hoped that some would have been able to attend, and the only conjecture hazarded as to their absence was that they were at that time wandering about in Camden Town in search of Airlie Gardens.

Seven midwives who are still in training at the East Ham Home were present, with their superintendent, Miss Rabson, and a number of friends and members of the committee, including Dr. Cullingworth.

Mrs. Bruce, in the course of the proceedings, said that the committee hoped to hold an annual gathering of past and present midwives, so as to keep in touch with those who had received their training and were at work. A badge was to be given to those who had done six months' work, who were then to become associates, and it was to be held by them as long as they continued to do work approved by the association; it would have to be relinquished on their giving up work. The motto on the badges expressed the spirit in which it was hoped the midwives would work.

Lady Balfour of Burleigh then presented Miss Rabson with a badge, and said that the Association were greatly indebted to her for her successful training of the midwives.

Miss Lucy Robinson, Vice-Chairman of the Executive Committee, assured the midwives present how much sympathy the committee felt for them, and especially when the question arose as to whether a doctor should be called in or not. They owed a great deal to all who sought to raise the standard of midwifery throughout the kingdom. She wished them all possible success in their work.

Lady Balfour of Burleigh having proposed a vote of thanks to Mrs. Bruce, some very excellent music was provided for the entertainment of the guests.

### Everybody's Opinion.

### THE DISTRICT NURSE AND PNEUMONIA.

"A HORSHAM NURSE" writes: May I criticise one suggestion in the Nurses' Clinic on "The District Nurse and Pneumonia," *re* linseed meal. My experience is that for large poultices it is much wiser to get the meal from a forage dealer or horse-keeper, where you can see it fresh ground—being used in such large quantities it is ground daily, whereas the chemist, especially in country districts, has so comparatively little demand for it and it comes to him already ground.

### "ARE NURSES UNDERPAID?"

MR. J. H. POLLITT writes from County Bank House, Blackburn: I beg the favour of a few words with reference to my speech at the recent meeting of the Royal National Pension Fund, and with reference to the observations thereon appearing in your pages. It would be well to try to arrive at an answer to the above question which would be generally accepted, and with that object I beg to suggest to your readers that it be gone into exhaustively in your columns, with your kind permission. The question really lies in small compass, and when we speak of nurses we must be understood to refer only to those who have been trained in some recognised training school. The highest type we can get are those from the middle classes who have been well educated and brought up in homes of refinement. These are the women the doctors and the public want, and the point for the public is whether the present pay will secure a continuous supply; or, to put it another way, to allow for the play of philanthropy on the part of nurses, is the public fairly treating its nurses in supporting them in such comfort as they have, and in making, or enabling them to make, such provision for old age as they do—namely, 8s. a week? That is the question, and it must be faced. In noting what is actually accomplished and in talking about possibilities we must have data, and I do not know of better data than are supplied by the reports of the Pension Fund. After nineteen years of work, and all praise to the Council of the Fund, there are 627 annuitants, with an average of £21. I shall expect some critics to say that these 627 nurses might have done better. Very good; let us have informed opinion on the point of how much better they could have done, and whether such reduction of personal expenditure would have caused injury to their health. This is important, and may involve efficiency.

### CAUTION TO ADVERTISERS.

MISS HELENA THOMPSON (President of the Workington District Nurses' Committee) writes as follows from Park End, Workington, under date of July 9: I enclose a cutting from the *Workington News* of June 16:—

WORKINGTON NURSE SENTENCED TO IMPRISONMENT.

"I AM VERY, VERY, SORRY."

The sequel to the arrest at Workington of Nurse Edith Isabel Gaskin, which created quite a sensation in the town, is contained in the *Nottingham Guardian* of the 13th of June, the report of the trial being as follows: At the Guildhall, Nottingham, yesterday (before Mr. W. L. Hardstaff and Mr. J. H. Bradwell), Edith Isabel Gaskin, nurse, described as of Mixbury, Northants, was charged with obtaining by false pretences on November 13th, a pair of shoes valued at 16s. 9d., the property of Messrs. Jessop and Sons, drapers, of King Street, Nottingham. Mr. F. Jackson prosecuted, and Mr. P. H. Buglass was for the defence. It was stated for the prosecution that prisoner, who was not in uniform, obtained a pair of shoes, saying they were to be entered to Miss Sullivan, at the Basford Sanatorium. Miss Sullivan was then a customer of the firm. The shoes had never been paid for, though prisoner left another pair to be mended. George Gibson, head gardener at the Sanatorium, stated that prisoner was formerly a nurse there, being known as Nurse Gaskin. Detective-officer Powdrill said that on the 30th ult., he received prisoner into custody at Workington, and when charged she admitted the offence, saying, "I am very, very sorry." Mr. Burgess appealed to the Bench for leniency, having regard to his client's position and capabilities. It was difficult to understand why she was guilty of such an act, but he felt certain that if dealt with leniently she would never repeat it. The Chairman said the Bench had decided to give prisoner another chance. She would have to go to prison for a month.

As Edith Isabel Gaskin will now be out of prison, I think it might be advisable if you could warn your advertisers against her. In February last we advertised for a district nurse in THE HOSPITAL. E. Gaskin applied for the situation, saying she had been for the last two years in that capacity at Bexhill-on-Sea, and giving as a reference a lady whom she represented as the Secretary of the Committee there, and we received a most excellent character. After she had been with us about three months we found the whole story to be a fabrication, and that she had written the character herself in the name of a lady who keeps a boarding-house there, and with whom she had lived since last December as a sort of help. The house had both a name and a number, so we never suspected they were both the same place. The lady alleged that E. Gaskin robbed her of £19 in money, as well as articles of jewellery, linen, and other things. She was arrested here on a warrant from Nottingham. On applying to us she showed a copy of a certificate of three years' training at Banbury Hospital; but after her arrest we inquired there, and found she was unknown to them. She pleased her patients, and evidently had some knowledge of nursing, though she was described in the Nottingham papers as a lace maker. Her age is about 30, rather small, slight, and fair. It is a pity that others should be deceived as we have been, and it would be well if you could warn your advertisers against her.

### Appointments.

British Lying-in Hospital.—Miss Alice Park, home sister, Guy's Hospital, has been appointed matron of the British Lying-in Hospital, York Road. Miss Park commenced her training in 1898, and has held the post of surgical sister at a nursing home, and sister of Queen Victoria Wards, the obstetric and gynæcological wards, at Guy's Hospital.

Dufferin and Mayo Hospital, Lahore.—Miss Lilian Tippetts, assistant matron, Guy's Hospital, has been appointed nursing superintendent of the Dufferin and Mayo Hospital, the Government Hospital, at Lahore. Miss Tippetts commenced her training as a probationer at Guy's in 1896, and has held the posts of staff nurse, night sister, out-patient sister, and home sister. Miss Tippetts also joined the A.N.S.R., and was one of the first to be sent out to South Africa during the Boer War. Miss Tippetts is also taking out to Lahore with her as sisters two Guy's sisters and a nurse. Miss H. Newton, sister of Lydia, a women's surgical ward; Miss Gertrude Morrah, sister of Charity Ward, also a women's surgical ward; and Miss E. R. Smith (who has just completed her three years' training, and has been head nurse), in Victoria, the obstetric ward, and Addison, a male medical ward.

East Ham Convalescent Home for Scarlet Fever.—Miss Bertha May has been appointed matron. She was trained at St. Bartholomew's Hospital, London, and has since been night sister at the Chest Hospital, City Road, London; sister at the Alexandra Hospital for Hip Disease, London, where she also did assistant matron's work; and sister of the Enteric Block at the Isolation Hospital, East Ham.

Gloucestershire County Nursing Association.—Miss A. L. Blackburn has been appointed assistant superintendent and health lecturer. She was trained at Burnley Victoria Hospital; is a Queen's nurse; has been superintendent of Sisters' Settlement and Nurses' Home, Old Kent Road, London; and lecturer on nursing and hygiene at Burnley, under the County Council.

Gore Farm Hospital, Dartford.—Miss Lillian E. Fincke has been appointed charge nurse. She was trained at the Royal Albert Hospital, Devonport, and has since been sister at North Devon Infirmary, Barnstaple, and sister at the General Hospital, Ramsgate.

Grimsby and District Hospital.—Miss E. Gillespie has been appointed theatre and out-patient sister, and Miss E. A. Pidgeon sister of the male and female wards. Miss Gillespie was trained at the Durham County Hospital, and has since been sister of the male and female wards at Grimsby Hospital. Miss Pidgeon was trained at the Royal Cornwall Infirmary, Truro, and was afterwards sister of the male wards and theatre for two years. She has also been sister at the Children's Hospital, Derby.

Hammersmith Health Visitor.—Miss Egglestone has been appointed. She was trained in Edinburgh and at Crumpsall Infirmary, Manchester. She has since been health visitor at Croydon. She holds the certificate of the Royal Sanitary Institute.

Home for Epileptics, Maghull, near Liverpool.—Miss Alice Hulme has been appointed assistant matron. She was trained at the General Infirmary, Chester. She has since been charge nurse at Carnarvonshire and Anglesey Infirmary, Bangor; sister at the Isolation Hospital, Chester; and sister at St. Mary's Hospital, Manchester. She has also done district and private nursing, and holds the certificate of the Central Midwives Board.

Infectious Diseases Hospital, Sutton.—Miss Louie Caine has been appointed charge nurse. She was trained at the Blackburn Fever Hospital, where she has been assistant nurse.

Llanelly Hospital.—Miss S. C. Kenrick has been appointed staff nurse. She was trained at Manchester Royal Infirmary, and has since been staff nurse at Blackheath and Charlton Hospital. She has also done holiday duty at Dewsbury Infirmary.

Newark-on-Trent Hospital and Dispensary.—Miss J. Horton has been appointed lady superintendent. She was trained at the West London Hospital, Hammersmith, and has since been sister at the Newark Hospital. She holds the certificate of the Central Midwives Board.

Norfolk and Norwich Hospital.—Miss Wilkinson, Miss Hewett, and Miss Poulden have been appointed sisters. Miss Wilkinson was trained at Wolverhampton General Hospital, and has since been sister at the Guest Hospital, Dudley; she has also done private nursing. Miss Hewett was trained at the Metropolitan Hospital, Kingsland Road, London, where she has since been holiday sister. Miss Poulden was trained at Poplar Hospital. She has since been staff nurse at the National Hospital for the Paralysed and Epileptic, Queen Square, Bloomsbury, and has also been engaged in private nursing.

Nourse Joint Hospital, Denver, Johannesburg.—Miss Fanny Wraith has been appointed matron. She was trained at the Fleming Memorial Hospital for Children, Newcastle-on-Tyne, and at Leeds General Infirmary. She has since been sister at the Lady Dudley Nursing Home, Johannesburg.

Nurses' Hostel Company, Limited.—Miss B. Chamberlain has been appointed secretary and assistant matron and takes up her duties on October 1. She was trained at St. Thomas's Hospital, and has since been a Queen's nurse, matron's assistant at "The Nurses' Co-operation," and sister-matron at the Royal Ear Hospital, Soho, London.

Royal Infirmary, Bradford.—Miss Ethel G. Massey has been appointed assistant matron. She was trained at the North Staffordshire Infirmary, Stoke-on-Trent. She has since been sister at the Royal Infirmary, Derby, ward sister and assistant matron at East Dulwich Infirmary.

St. Mark's Hospital, City Road, London.—Miss Frances Sherriff has been appointed night sister. She was trained at St. Mark's Hospital, and previous to training was in a private nursing home at Weymouth. She holds the certificate of the Central Midwives Board.

St. Mary Islington, Infirmary.—Miss G. A. Line has been appointed staff nurse. She was trained at St. Mary Islington, Infirmary, and previous to her training was employed at Stepney Infirmary, Bromley by Bow, and Holborn Infirmary, Shepherdess Walk, City Road, London.

Shipston-on-Stour Cottage Hospital.—Miss Locke has has been appointed nurse-matron. She was trained at St. Bartholomew's Hospital, London, and St. Pancras Infirmary; and for maternity at Queen Charlotte's Hospital, London. She has been charge nurse at Mansfield Sanatorium, and staff nurse at Brompton Hospital. She has also held other appointments.

Walsall and District Hospital.—Miss Gaved Wills has been appointed matron. She was trained at the South Devon and East Cornwall Hospital, Plymouth, and has since been charge nurse at St. Peter's Hospital, Covent Garden, London; night superintendent and day sister at the West London Hospital, Hammersmith; and for the past four years lady superintendent of the Hospital and Dispensary, Newark-on-Trent.

### "Cheer Up" Column.

"We must laugh before we are happy,
For fear we die, before we laugh at all."

CHICAGO TINNED MEATS.

Some one wrote to the packers, "What sin
To put a man's head in the tin."
They said, "It's absurd
To say it occurred;
Nothing larger than thumbs has gone in."

ADAPTABILITY.

I read recently of the adaptability of a nurse, the figure of which is not much overdrawn. A patient in a hospital to his pretty nurse said, "Will you marry me?" The nurse said, "Certainly." The patient then said, "Then do you love me?" She replied, "Oh no, that is merely a part of the treatment. I must keep my patients cheerful. I promised this morning to run away with a married man who had lost both his legs."—*Dr. B. A. Wilks.*

APRONS AND APPENDICITIS.

Mr. Smith came home from business the other evening and said to Mrs. Smith: "I think I am going to have appendicitis."

Mrs. Smith's reply finished him: "I think I am going to have a new spring bonnet, Tom, so your appendicitis will have to wait."

GOSSIPS.

"I knew a feller once that had
The yeller jaunders awful bad, and
Each and every one he'd meet, would stop and
Give him some receet for curin' of them.
But, he'd say, he kinder guessed they'd go away,
Without no medicine, and boast
That he'd get well without one dost.
And so he kept a yellerin' on, and they
Predictin' that he'd die some day,
Before he know'd it. Tuk his bed
The feller did, and lost his head.
And wandered in his mind a spell,
And rallied and at last got well.
But everyone that said he'd die
Went back on him eternally."—*J. W. Riley.*

SELF-PRESERVATION.

The surgeon gave instructions to a student to set a fractured leg.

Next day the surgeon, on examining the patient, said to him: "What a fool you must be. Why did you let him set the sound leg?"

The patient replied: "If he could not tell my broken leg from t'other, should not I have been a fool to let him set the broken?"

THAT HEAD OF HAIR!

At a cosmopolitan hospital the staff are proud of their mastery of many tongues. A gentleman of colour came to the casualty room the other day with a magnificent head of hair, but what language he spoke, or what he wanted, no one could tell. A careful examination revealed no malady, but there was the patient to be dealt with by the staff. After earnest consultation he was ordered a hot bath, to have his hair cut, and be put to bed. Next morning it was discovered that the gentleman of colour had called at the hospital to visit a friend of his in one of the wards!

(*Contributions for this column are invited, and, if accepted, will be paid for.*)

### Presentations.

LINCOLN NURSING INSTITUTION.—Miss Bromhead, Superintendent of the Lincoln Nursing Institution, was presented last Thursday with an illuminated address from 115 present and past nurses of her staff congratulating her upon the completion of forty years of work in the nursing field, and expressing their high esteem for her.

ROYSTON COTTAGE HOSPITAL.—On leaving the Royston Cottage Hospital, Sister Dandison was the recipient of many kind tokens of gratitude not only from those who have been inmates of the institution, but also from visitors and friends of patients, all of whom expressed universal regret at her departure. The Committee, while thanking Miss Dandison for the good work done during her stay at the institution, presented her with a cheque for £20 as a mark of respect.

### The Nurses' Bookshelf.

HANDBOOK OF OBSTETRIC NURSING. By FRANCIS W. N. HAULTAIN, M.D., F.R.C.P.Edin., and JAMES HAIG FERGUSON, M.D., F.R.C.P.Edin., M.R.C.S.Eng. (Edinburgh and London: Young J. Pentland. Fifth edition. 1906. Pp. 266. 37 illus. Price 5s.)

THERE is little room for criticism in this, the fifth, edition of Haultain and Ferguson's handbook of obstetric nursing. From the introduction, with its simple, rapid survey of the anatomy and physiology of the body, to the excellent glossary and index at the end, it is full of concise, useful information, singularly free from circumlocution, and, by means both of letterpress and illustration, conveying it in the most succinct manner to the reader. No nurse, after carefully perusing this book, could have the slightest doubt remaining as to the importance of antiseptics in obstetric work, and a deep sense of her own responsibilities with regard to it. A chapter devoted to a "Description of certain Methods and Appliances" is full of minute directions for the proper carrying out of her duties in making a vaginal examination, giving an enema or suppository, vaginal douching and plugging, passing the catheter, bandaging the breasts, abdomen, and vulva, and the use of the breast-pump, hypodermic syringe, etc. Continually is the necessity for surgical cleanliness insisted on in every detail, yet not too often to ensure its being properly carried out, as it is indeed the essence of success in all obstetric work. A chapter is devoted to the "Management of the Child," with general rules for feeding it, if artificial means have to be used. There is also a brief account of most of the ailments incidental to early infancy, with directions as to their treatment. The use of the so-called "comforter" is tolerated, if not advised, especially for "delicate children," but on this point opinion is strongly divided, most medical men being greatly opposed to employing one in any circumstances whatever.

### TRAVEL NOTES AND QUERIES.

BY OUR TRAVEL CORRESPONDENT.

AUSTRIAN TYROL DOLOMITES.—Owing to the number of applicants asking for information about the Dolomite Tour, and wishing to go later in the summer, Miss L. M. Davidson has decided to make another on the same lines and covering most of the same ground, starting on or about August 17 or 20, 1906. The route will be Boulogne or Calais, Laon, Bâle, Zurich, Innsbrück, Toblach, Tre Croce, Cortina, thence through some of the finest Dolomite scenery—Franzensfeste, Innsbrück, Bale, London. Members may join for a fortnight, three weeks, twenty-five days, or four weeks. All applications should be addressed to Miss L. M. Davidson, 25 Marjorie Grove, Lavender Hill, London, S.W.

### Notes and Queries.

**REGULATIONS.**

**The Editor is always willing to answer in this column, without any fee, all reasonable questions, as soon as possible.**

**But the following rules must be carefully observed.**

**1. Every communication must be accompanied by the name and address of the writer.**

**2. The question must always bear upon nursing, directly or indirectly.**

**If an answer is required by letter a fee of half-a-crown must be enclosed with the note containing the inquiry.**

*Addresses Wanted.*

(183) Can you give me the address of the (1) New British Red Cross Society, and (2) the Nurses' Institute for the Hop-pickers?—*Nil Desperandum.*

(1) The National Society for Aid to Sick and Wounded in War has its office at 5 York Buildings, Adelphi, W.C. (2) With regard to the Hop-pickers, see the note in this week's issue headed, "The Hop-picking Season."

*Coca.*

(184) Is coca the same drug as that in coca wine, and if used in a medicine with other ingredients, is it injurious to the stomach?—*Auntie Za.*

Yes, coca is present in coca wine. Coca is not a drug which an amateur should prescribe, and a physician should certainly be referred to before using it.

*Private Maternity Homes.*

(185) Can you tell me of some private maternity homes where I can get constant employment, and is general training required, or is a maternity certificate sufficient?—*Nurse L.*

We cannot do as you ask, but you will find the addresses of maternity homes in "Medical Homes," price 7d., from The Scientific Press, 28 Southampton Street, Strand. General training is a recommendation, but it is not always required.

*Paralysis.*

(186) I have a relative who is paralysed. She now suffers from hæmorrhage. Is this usual?—*Dorothy.*

You had better consult a medical man.

*Up Country Nursing Association.*

(187) What is the address of the above?—*Mary.*

The new address is 68 Regent's Park Road, N.W.

*A Probationer's Age.*

(188) Will you tell me if, at 36 years of age, I can be accepted as a probationer in any hospital?—*Provincial.*

Consult "How to Become a Nurse," published by the Scientific Press, 28 Southampton Street, Strand; but we are afraid that you will find it difficult to gain admission. You might, however, advertise with success.

*A Diet for Gout.*

(189) Can you tell me in what book I shall find information for the diet of a patient suffering from gout and an abscess in his lung?—*Cantharides.*

Have you not consulted his doctor? "Diet in Sickness and Disease," by Mrs. Ernest Hart, The Scientific Press, 28 Southampton Street, Strand, may help you.

*A Remedy Wanted.*

(190) Can you tell me of a remedy for the complaint I mention in my letter?—*Nurse May.*

If your medical man has failed to help you, we fear we cannot recommend anything.

*A Hospital in London.*

(191) Can you tell me if any hospital in London treats bad legs?—*N. W.*

You appear to be a nurse, and, if so, you should write to the Secretary of any hospital in London, and doubtless your case will receive attention. But as there are excellent hospitals in the city you write from, why not apply to one, as probably you would be accepted only as an out-patient in London?

**Handbooks for Nurses.**

| | Post Free. |
|---|---|
| "How to Become a Nurse: How and Where to Train." | 2s. 4d. |
| "Nursing: its Theory and Practice." (Lewis.) ... | 3s. 6d. |
| "Nurses' Pronouncing Dictionary of Medical Terms." | 2s. 6d. |
| "Complete Handbook of Midwifery." (Watson.) ... | 6s. 4d. |
| "Preparation for Operation in Private Houses." ... | 0s. 6d. |

**Of all booksellers or of The Scientific Press, Limited, 28 & 29 Southampton Street, Strand, London, W.C.**

### For Reading to the Sick.

THE BOW IN THE CLOUD.

A fragment of a rainbow bright,
 Through the moist air I see,
All dark and damp on yonder height,
 All clear and gay to me.

An hour ago the storm was here,
 The gleam was far behind,
So will our joys and griefs appear
 When earth has ceased to blind.

Grief will be joy, if on its edge
 Fall soft that holiest ray:
Joy will be grief, if no faint pledge
 Be there of heavenly day.

*John Keble.*

Why did Jesus lead the deaf man aside? His purpose was, that apart from the din and tumult and interruptions of the crowd, in solitude and silence, the man might be a recipient of deep and lasting impressions; even as the same Lord does now oftentimes lead a soul apart, sets it in the solitude of a sick-chamber, or in loneliness of spirit, or takes away from it earthly companions and friends, when He would speak with it and heal it.—*Archbishop Trench.*

Blessed, then, is sickness or sorrow or any experience that compels us to stop, that takes the work out of our hands for a little season, that empties our hearts of their thousand cares, and turns them toward God to be taught of Him.

But why should we wait for sickness or sorrow to compel into our lives these necessary quiet hours? Would it not be far better for us to train ourselves to go apart each day for a little season from the noisy, chilling world, to look into God's face and into our own hearts, to learn the things we need so much to learn, and to draw secret strength and life from the fountain of life in God.—*Dr. Miller.*

Love gladly suffereth for one whom it loveth. It joys to suffer in proof of its love. Love sweetens all bitter things, softens all hard words, smooths all which is toilsome, makes fasting a feast, self-denial for Christ's poor a joy; labour, rest; and rest out of God, weariness; waking early with Christ, refreshment. Bodily pain is hallowed to it by His Cross, and it receives each throb or pang from its loving Father's Hand, as distilling like the dew upon it from that precious cup which He for our sakes gave to His well-beloved Son.—*E. B. P.*

O Lord our God, under the shadow of Thy wings let us hope. Thou wilt support us, both when little, and even to gray hairs. When our strength is of Thee, it is strength; but, when our own, it is feebleness. We return unto Thee, O Lord, that from their weariness our souls may rise towards Thee, leaning on the things which Thou hast created, and passing on to Thyself, who hast wonderfully made them; for with Thee is refreshment and true strength. Amen.—*St. Augustine.*

## Figures and Tables

**Figure f1:**